# The Evaluation of New-Generation Biomarker sCD14ST Provides New Insight into COVID-19’s Effect on Bone Remodeling

**DOI:** 10.3390/jcm14030979

**Published:** 2025-02-03

**Authors:** Emanuela Galliera, Luca Massaccesi, Laura Mangiavini, Elena De Vecchi, Francesca Villa, Massimiliano Marco Corsi Romanelli, Giuseppe Maria Peretti

**Affiliations:** 1 IRCCS Ospedale Galeazzi-Sant’Ambrogio, 20157 Milan, Italy; luca.massaccesi@unimi.it (L.M.); laura.mangiavini@unimi.it (L.M.); elena.devecchi@grupposandonato.it (E.D.V.); francesca.villa@grupposandonato.it (F.V.); giuseppe.peretti@unimi.it (G.M.P.); 2 Department of Biomedical Sciences for Health, Università degli Studi di Milano, 20133 Milan, Italy; mmcorsi@unimi.it; 3 Department of Experimental and Clinical Pathology, IRCCS Istituto Auxologico Italiano, 20095 Cusano Milanino, Italy

**Keywords:** COVID-19, sCD14ST, bone remodeling, osteoimmunological biomarkers

## Abstract

**Background/Objectives**: The COVID-19 pandemic has increased interest in osteoimmunology because of the impact of SARS-CoV-2 on both the immune system and the bone microenvironment. Soluble CD14ST could influence the production of the osteoimmunological regulators of osteoclast differentiation. The aim of this study is to evaluate the role of sCD14ST in COVID-19’s effects on bone remodeling—evaluating, in particular, the correlation with new-generation osteoimmunological biomarkers—and to acquire comprehensive knowledge of the effects of the disease on the immune and skeletal system. **Methods**: The serum level of sCD14ST was measured in COVID-19-positive and COVID-19-negative patients undergoing orthopedic surgery and correlated with the inflammatory and osteoimmunological biomarkers RANKL/OPG, FGF23, IL-6, C-reactive protein (CRP), procalcitonin (PCT), sRAGE, and SuPAR. **Results**: In our patients, sCD14ST showed a strong increase in COVID-19-positive patients, and a significant decrease in tandem with the infection resolution, confirming its diagnostic and prognostic value. sCD14ST was more clinically relevant than the two canonically inflammatory makers used in the clinical protocols, CRP and PCT, and displayed a good positive correlation with FGF23, RANKL/OPG, IL-6, and SuPAR and a negative correlation with sRAGE. **Conclusions**: Monitoring sCD14ST along with SuPAR may offer valuable insights into immune system dysregulation and bone-related complications in conditions characterized by inflammation. These soluble receptors represent important links between immune activation and bone metabolism, especially in the context of diseases like COVID-19, where the inflammatory response may impact bone fragility.

## 1. Introduction

The lack of reliable biomarkers for COVID-19 has been a serious challenge in the managing and understanding of this disease. For COVID-19, moreover, there is still a lack of consistent biomarkers that can predict disease severity, progression, and long-term outcomes [1,2]. Recent evidence from our group suggested a potential diagnostic and prognostic role of the biomarker sCD14ST [3] in the prediction of COVID-19 outcomes. sCD14-ST is a soluble form of a glycoprotein expressed on monocytes and macrophages, released into circulation in response to pro-inflammatory signals. It is described as a useful marker for early diagnosis, risk stratification, and prognosis prediction in several infections [4,5,6]. The biomarker sCD14ST was suggested as a useful prognostic tool with which to predict SARS-CoV-2 outcomes in association with new-generation makers, such as SuPAR (soluble urokinase plasminogen activator receptor) [2,3]. SuPAR is a soluble molecule that can easily be measured in plasma and serum, reflecting the level of immune system activation [7,8], and recent evidence has suggested its potential role as a predictor of COVID-19 outcomes [9,10,11]. One of the challenging aspects of COVID-19 is its complex disease mechanism; COVID-19 can affect different organs, making it hard to identify biomarkers that capture the full effect of the disease [12,13]. COVID-19 primarily affects the respiratory system; however, little is known about the extrapulmonary effect of COVID-19, particularly on the bone system. Recent evidence has indicated a relationship between COVID-19 and bone health [14,15], affecting bone density, bone loss, and the risk of fractures [16,17,18,19] Severe COVID-19 disease is characterized by a dysregulated immune response, including an overactive inflammatory cascade, which can impact bone remodeling. The interactions between the immune and skeletal systems are described by a new interdisciplinary field called osteoimmunology, describing how immune cells influence bone remodeling, health, and disease, and how bone tissue affects immune responses [20,21,22]. The COVID-19 pandemic has increased interest in osteoimmunology because of the impact of SARS-CoV-2 on both the immune system and the bone microenvironment. Osteoimmunological molecules have been described as potential biomarkers of bone remodeling indicators in inflammatory conditions [23,24,25,26,27,28], but their potential role in evaluating the effect of COVID-19 on bone fragility has only been recently described [29]. In particular, the osteoimmunological biomarker RANKL/OPG ratio has been suggested as an improvement in the clinical evaluation of COVID-19’s effect on bone loss [30,31]. Soluble CD14ST could influence the production of RANKL or OPG, which are osteoimmunological regulators of osteoclast differentiation [32]. There is emerging evidence that soluble sCD14St plays a role in bone metabolism, particularly in inflammatory bone diseases. sCD14ST may act as a biomarker for disease activity, reflecting levels of immune activation that could stimulate osteoclastogenesis through pro-inflammatory cytokine release, but the specific role of sCD14ST on COVID-19-induced effects on bone loss remains unexplored. The aim of this study is to evaluate the role of sCD14ST in COVID-19’s effects on bone remodeling—evaluating, in particular, its correlation with new-generation osteoimmunological biomarkers—and to acquire comprehensive knowledge of the effect of the disease on the immune and skeletal system. This is, to our knowledge, the first study evaluating sCD14ST and osteoimmunological biomarkers at the same time with respect to COVID-19’s effect on bone metabolism; with SCD14ST being involved in osteoimmunology and a marker of COVID-19 outcome, this diagnostic approach could provide simultaneous information on both the infection and the effect on bone turnover. In this way, the evaluation of CD14ST could provide new insight into diagnostic and therapeutic approaches to COVID-19.

## 2. Materials and Methods

### 2.1. Study Design and Patient Enrollment

This was an observational single-center study carried out from April 2021 to April 2023. The study enrolled patients admitted to the IRCCS Galeazzi Orthopedic Institute for fracture, and they were divided into two groups, namely, patients with COVID-19 infection and patients without COVID-19, both confirmed via nasopharyngeal swab during the patients’ clinical routine. Real-time PCR on a nasopharyngeal swab specimen was performed for the diagnosis of SARS-CoV-2 infection.

A total of 45 patients (12 males and 33 females; age 80.25 ± 10.84 yrs) were initially enrolled. Then, 10 patients were ruled out for lacking a COVID-19 infection diagnosis, so only the remaining 35 patients (11 males and 24 females; age 80.51 ± 11.39 yrs) were analyzed in the study. Sample size was calculated using a dedicated statistical program, considering a type-1 and type-2 error rate of 0.05 and 0.4, respectively, and considering an effect size of 0.85 and a standard deviation of 1.

The inclusion criteria were as follows: patients of both genders with a fracture of the proximal femur; patients age 50 years or more; signature of informed consent; pathologies linked to bone fragility (fractures, osteoporosis, parathyroid disease); having performed a nasopharyngeal swab to verify SARS-CoV-2 infection. Exclusion criteria were as follows: autoimmune diseases; hypovitaminosis D; steroid therapy; or therapy that can affect the inflammatory response.

This analysis was performed on residual blood samples obtained for the normal diagnostic flow. On all the patients, radiographic investigations, as laboratory tests required by clinical routine, were performed at different time points: T0 (pre-operative); T1 (24 h post-operative); T2 (3 ± 1 days post-operative).

All research related to human use complied with all the relevant national regulations and institutional policies and was conducted in accordance with the Helsinki Declaration. Informed consent was obtained from all participants. This study was approved by the local ethics committee [CE of IRCCS San Raffaele Hospital, Milan, CE 18/INT/2021]. Details that might disclose the identity of patients under study were omitted, in accordance with HIPAA. This study was registered as COVID BONE [NCT05352295] to ClinicalTrials.gov.

### 2.2. Blood Sample Collection and Serum Preparation

Blood drawing was performed on all patients, and serum and plasma EDTA were obtained at all time points. Serum samples were collected after whole-blood collection, clotting, and centrifugation at 400× *g* for 10 min at RT without braking. The undiluted serum was aliquoted and stored in polypropylene tubes. Plasma + EDTA and serum samples were stored at −80 °C.

### 2.3. Evaluation of sCD14-ST

sCD14-ST concentration (pg/mL), IL-6 concentration (pg/mL), and procalcitonin (PCT) concentration [ng/mL] were measured using CL-1200i (Mindray, Shenzen, China) according to the manufacturer’s protocol based on the sandwich immunoenzymatic assay (CLIA) using monoclonal antibodies for sCD14-ST, IL-6, and PCT. CL-1200i is a floor-standing, fully automatic, chemiluminescence immunoassay system offering up to 240 tests per hour using the ALP-AMPPD principle, as previously described [30].

### 2.4. Quantification of SuPAR and Osteoimmunological Markers

SuPAR was measured via SuPARnostic ELISA Assay according to manufacturer’s protocol (Birkerød, Virogates, Denmark). RANKL and OPG were measured using an ELISA sandwich Quantikine Assay according to manufacturer’s protocol (Pikokine TM ELISA for OPG; quantitative sandwich ELISA for RANKL and FGF23, MyBioSource, San Diego, CA, USA). sRAGE was measured using an ELISA sandwich Quantikine Assay according to manufacturer’s protocol (R&D System, Minneapolis, MN, USA). C-Reactive protein (CRP) was measured using immunoturbidimetry on an automated biochemical analyzer (Olympus CRP-Latex assay, Central Valley, PA, USA).

## 3. Results

### 3.1. Evaluation of sCD14ST in COVID-19-Positive and COVID-19-Negative Patients

**Figure 1:**
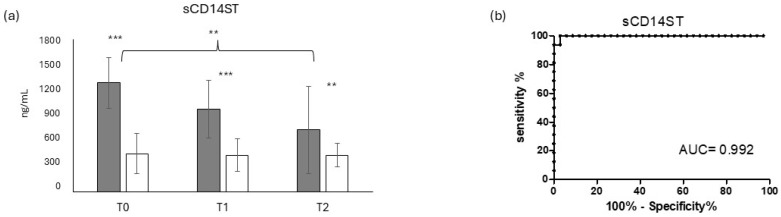
Evaluation of sCD14ST in COVID-19-positive and COVID-19-negative patients. Longitudinal evaluation of sCD14St in COVID-19-positive (dark grey bars) and COVID-19-negative (light grey bars) patients (**a**) and the relative ROC (receiving operating curve) (**b**); ** = *p* < 0.01, very significant; *** = *p* < 0.001, extremely significant.

Figure 1a shows the circulating level of sCD14-ST in COVID-19-positive (dark grey bars) and COVID-19-negative (light grey bars) patients. The biomarker sCD14-ST displayed very high circulating levels in COVID-19-positive patients at T0, with a very significant difference compared to COVID-19-negative patients (*p* < 0.001). This significant difference was maintained along the time points T1 and T2, even though sCD14-ST showed a progressive decrease over time. At the early time point, T1, the decrease in sCD14-ST was not statistically significant, while at T2, it displayed a quite statistically significant decrease compared to T0 (*p* < 0.01). In COVID-19-negative patients, sCD14-ST showed very low and constant level. The striking difference between COVID-19-positive and COVID-19-negative patients was confirmed by the very high ROC (receiver operating curve) AUC (area under the curve = 0.992), as shown in Figure 1b.

### 3.2. Correlation Between sCD14ST and Markers of Inflammation

**Figure 2:**
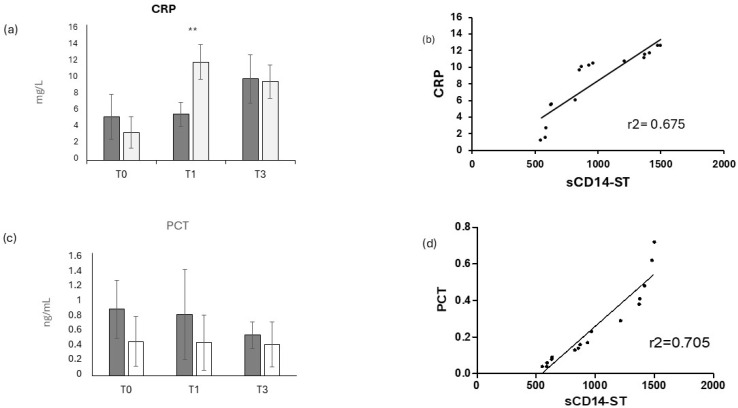
Inflammatory biomarkers’ longitudinal evaluation. Longitudinal evaluation of CRP (**a**) and PCT (**c**) in COVID-19-positive (dark grey bars) and COVID-19-negative (light grey bars) patients, and the relative ROC (receiving operating curve) for CRP (**b**) and PCT (**d**), respectively; ** = *p* < 0.01, very significant.

In order to evaluate the inflammatory response in COVID-19-positive and COVID-19- negative patients, two canonical markers of inflammation, CRP and PCT, were measured, as shown in Figure 2. CRP displayed no significant differences either between the two groups of patients or in the longitudinal evaluation, as previously reported [30]. This result is consistent with the weak correlation (Figure 2b) with sCD14-ST (r^2^ = 0.675). Similarly, PCT was measured in COVID-19-positive and COVID-19-negative patients, and it displayed a weak but not statistically significantly higher level in COVID-19-positive patients compared to negative ones (Figure 2c) at all time points, with a mild but not significant decrease over time. Consistently, PCT showed a weak correlation with sCD14-ST (r^2^ = 0.705), as shown in Figure 2d.

### 3.3. Correlation Between sCD14ST and Osteoimmunological Markers

**Figure 3:**
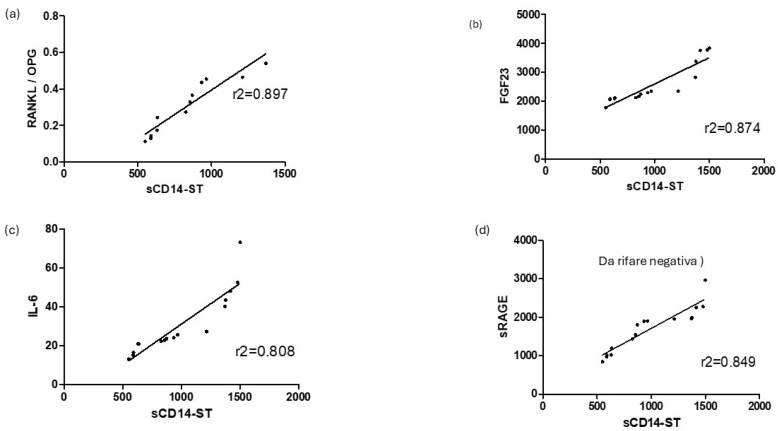
Correlation of sCD14ST with osteoimmunological biomarkers. sCD14ST linear regression analysis correlation with RANKL/OPG (**a**), FGF23 (**b**), IL-6 (**c**), and sRAGE (**d**).

The biomarker sCD14-ST was correlated with the osteoimmunological biomarkers RANKL/OPG, FGF23, IL-6, and sRAGE, as shown in Figure 3. The circulating concentration of this biomarker has been previously reported [30]. sCD14ST displayed a good correlation with RANKL/OPG (r^2^ = 0.897), an osteoimmunological marker of bone resorption. Consistently, sCD14ST displayed a good correlation with FGF23 (r^2^ = 0.874), a biomarker of bone fragility. The inflammatory cytokine IL-6 showed a good correlation with sCD14ST (r^2^ = 0.808). Conversely, sCD14ST displayed a negative correlation with the inflammatory prognostic biomarker sRAGE (r^2^ = −0.924).

### 3.4. Evaluation of SuPAR in COVID-19-Positive and COVID-19-Negative Patients and Correlation with sCD14ST and Osteoimmunological Biomakers

**Figure 4:**
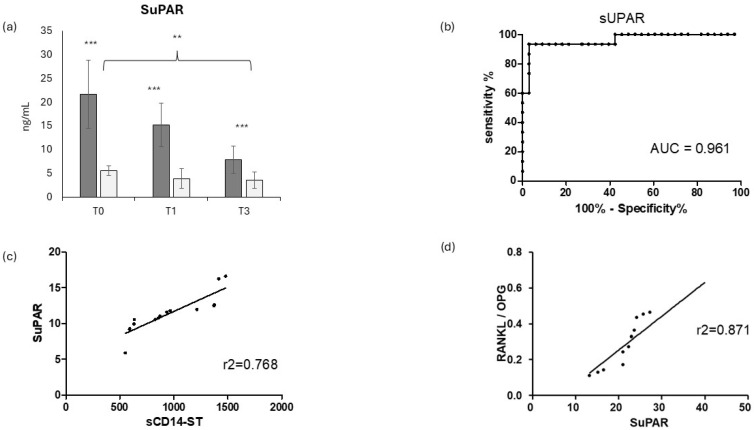
SuPAR evaluation: longitudinal evaluation of SuPAR in COVID-positive (dark grey bars) and COVID-negative (light grey bars) patients (**a**) (** = *p* < 0.01, very significant; *** = *p* < 0.001, extremely significant) and the relative ROC (receiving operating curve) (**b**). SuPAR linear regression analysis correlation with sCD14St (**c**) and RANKL/OPG ratio (**d**).

Since the canonical biomarkers of inflammation CRP and PCT were not informative in this study, an alternative and new infection biomarker, SuPAR (soluble urokinase plasminogen activator receptor) was evaluated, as shown in Figure 4. Similarly to sCD14ST, SuPAR displayed a significantly higher level in COVID-19-positive patients at all time points (*p* < 0.001) (Figure 4a), with a significant decrease over time (*p* < 0.01, comparing T2 and T0). This strong increase in COVID-19-positive patients was confirmed by a high ROC AUC (0.961) (Figure 4b). Consistently, SuPAR displayed a good correlation with sCD14ST (r^2^ = 0.768) (Figure 4c). In order to evaluate whether SuPAR could be associated with sCD14ST in the evaluation of COVID-19-induced bone resorption, the correlation of SuPAR with RANK/OPG ratio was measured (r^2^ = 0.871) (Figure 4d).

## 4. Discussion

Biomarkers could help tailor treatments to patients based on their specific disease profiles. Without these, treatments tend to be more generalized and potentially less effective. The variety of symptoms in COVID-19 are diverse, and without biomarkers, it is challenging to diagnose, monitor, and develop treatments for these effects [33,34,35,36,37,38].

sCD14ST has been described as a good biomarker to predict the severity in COVID-19 [39,40], particularly in critically ill patients [41,42]. A recent study of our group showed the ability of sCD14ST to predict the outcome in intensive care unit patients [3]. In this study sCD14ST confirmed its diagnostic ability, evidenced by the highly significant increase in COVID-19-positive patients compared to negative ones at T0, and by the high AUC ROC curve. In the short longitudinal evaluation, sCD14ST showed a significant decrease in tandem with the infection resolution, confirming its prognostic value (Figure 1). In particular, sCD14 ST was more clinically relevant than the two canonically inflammatory makers used in the clinical protocols, i.e., CRP and PCT. At any time point, both these markers showed little to no difference between the two groups of patients (Figure 2). This result underlines how COVID-19 presentation can show a variety of symptoms, which can be challenging to detect with the canonical makers of inflammation, particularly in COVID-19 patients who do not present severe symptoms [43,44], as in our study. Consistently, these two biomarkers showed a quite-weak correlation with sCD14ST, having, on the contrary, a high diagnostic value.

While there is an increasing body of knowledge concerning the impact of COVID-19 on the lungs, heart [45,46], and other vital organs, there is increasing interest in its long-term consequences on bone health.

The COVID-19 pandemic has had a profound impact on several aspects of health, including dietary habits, physical activity, access to medical care, and, as a consequence, bone health and density. Sedentary lifestyles can also contribute to reducing bone density. During lockdown, the reduced access to fresh and healthy food options resulted in nutrient deficiencies, particularly in calcium and vitamin D, both of which are crucial for bone health. Moreover, many people delayed or avoided routine medical care during the pandemic, including bone density screenings, which could lead to underestimated or untreated bone health issues. All these aspects of the COVID-19 pandemic could have an impact on bone health. Indeed, bone density can be adversely affected by a combination of poor nutrition (especially low calcium and vitamin D intake), lack of physical activity, and reduced access to elderly people. The elderly, in particular, are already at a higher risk of bone density loss and are more susceptible to severe forms of COVID-19 infection; thus, they may have been particularly affected by these factors, leading to an increased risk of falls and fractures. Recent studies described the effect of COVID-19 on muscle wasting [47] and bone mineral density loss due to the inflammatory responses [48,49] sCD14ST, an inflammatory marker, may play a role in this process by contributing to the increased inflammatory response, which can affect bone metabolism.

The mechanism linking sCD14ST, inflammation, and bone health is still an area of active research. sCD14ST can enhance the effects of pro-inflammatory cytokines (like TNF-α, IL-1, and IL-6), which are involved in the differentiation and activation of osteoclasts, the cells responsible for bone resorption.

Increased levels of sCD14ST in circulation can indicate a heightened inflammatory state, which may lead to increased osteoclast activity and subsequent bone resorption. In addition, inflammatory cytokines, associated with high sCD14ST levels, can also impair osteoblast function and reduce bone formation. Recent evidence suggested that in COVID-19 patients, where there is significant immune activation, inflammation could exacerbate bone resorption [50]. In the context of COVID-19, osteoimmunology helps explain how immune system activation, inflammation, and treatments like corticosteroids contribute to bone loss [30,31,50]. Osteoimmunology is the study of the complex interactions between the immune system and bone metabolism, and it plays a significant role in understanding how diseases like COVID-19 can affect bone health. A recent study from our group showed the crucial role of osteoimmunological biomarkers in the evaluation of COVID-19 [29], describing a significant role for the RANKL/OPG ratio (the osteoimmunological markers of bone resorption), the bone fragility marker FGF23, and the interaction with two inflammatory markers: the primary cytokine IL-6 and the soluble maker sRAGE. In order to evaluate whether sCD14ST can only provide information on COVID-19 infection or whether it can also provide information on inflammatory-induced bone loss, in this study, circulating sCD14ST levels were correlated with the previously mentioned osteoimmunological biomarkers (Figure 3). The present study showed a strong correlation of sCD14ST with the main osteoimmunological biomarker of bone resorption: the RANKL/OPG ratio. The RANKL/OPG ratio represents the balance between the bone-resorbing [RANKL] and bone-protecting (OPG) factors. It is reported that in inflammatory conditions like COVID-19, immune activation can affect the RANKL/OPG ratio [30]. Elevated levels of inflammatory cytokines (such as IL-6, TNF-α, and IL-1β) can increase the production of RANKL, leading to a higher RANKL/OPG ratio and increased osteoclast activity [51,52,53]. This result is consistent with previous evidence showing a correlation between sCD14ST and RANKL/OPG in the context of osteomyelitis, a bone infection leading to bone loss [32]. The strong correlation of sCD14ST with RANKL/OPG in COVID-19 patients suggests that sCD14ST could also be helpful in the context of COVID-19, providing useful insight into COVID-19-induced bone loss. This result is also supported by the strong correlation of sCD14ST in COVID-19 patients with the bone fragility biomarker FGF23. FGF23 has been shown to be involved in COVID-19-induced bone loss [29,52,53,54,55]. Taken together, these results suggest that sCD14ST could provide direct and useful insights into COVID-19 outcomes not only with respect to the inflammatory response but also with respect to COVID-19-induced bone loss. In order to complete the inflammatory response evaluation, sCD14ST was also correlated with the primary cytokine IL-6, one of the main mediators of the COVID-19-induced “cytokine storm” [56]. IL-6 acts as a major player in the systemic effect of the pro-inflammatory acute inflammatory response, and it has recently been described as a COVID-19 severity predictor [57]. sCD14ST showed a very good correlation with IL-6, confirming its value as a biomarker of the COVID-19-induced inflammatory response. The COVID-19-induced inflammatory response can also result in an increase in reactive oxidative species (ROS), which may cause protein damage, as well as an increase in advanced glycation end-products (AGEs). RAGE is a membrane-bound receptor, and its soluble form, sRAGE, acts as a decoy receptor and disease biomarker [58]. RAGE was also recently reported to be directly involved in COVID-19 as a marker of disease outcome [59,60]. AGEs and RAGE signaling have been implicated in the pathogenesis of osteoporosis and bone loss [59,60]. AGEs can accumulate in bone tissue over time, promoting oxidative stress, inflammation, and increased bone resorption. sRAGE plays a role in mitigating inflammation by binding AGEs, and lower levels of sRAGE in COVID-19 patients may indicate an excessive inflammatory response [59]. Therefore, in conditions where sRAGE levels are low or where AGE accumulation is high, bone health may be compromised. Both sCD14ST and sRAGE offer insights into immune system function and inflammation and may have implications for bone health and systemic diseases. In our COVID-19 patients, sRAGE displayed a significative reduction from a high level over time. Consistently, sCD14ST showed a negative correlation with sRAGE. Monitoring these markers may help in understanding and managing bone complications in diseases like COVID-19. Evidence from bone infection, such as osteomyelitis or prosthetic joint infection (PJI), has suggested another biomarker, along with sCD14ST, able to conjugate inflammatory response and bone loss [60]: SuPAR [soluble urokinase plasminogen activator receptor]. SuPAR is a soluble molecule that can be easily measured in plasma and serum, reflecting the level of immune system activation. SuPAR is involved in leukocyte recruitment and coagulation in the inflammatory response to infection [61,62]. This biomarker is well recognized as a prognostic factor in different kinds of infections and correlates with the severity of disease [6,8,9]. Since SuPAR has been described as a diagnostic and prognostic biomarker in the case of inflammation and bone infection, the correlation with sCD14ST was evaluated in this study. SuPAR displayed a strong difference between COVID-19-positive and COVID-19-negative patients (Figure 4a), confirming good diagnostic and prognostic values, as indicated by the high ROC AUC (Figure 4b).

sCD14ST showed a positive correlation with SuPAR, reinforcing the link between these two biomarkers of immune activation and inflammation. Both molecules are indeed elevated during inflammatory responses and have been individually described as biomarkers of disease severity in conditions like COVID-19.

While SuPAR itself is not directly involved in bone remodeling, elevated levels of SuPAR can signal systemic inflammation, which can impact bone health. In diseases like COVID-19, sCD14ST and SuPAR can be used to track disease severity and inflammation levels [11,43]. Elevated levels of SuPAR and sCD14ST are indicative of more severe immune activation, which can influence the RANKL/OPG axis and lead to bone-related complications. For this reason, the correlation between SuPAR and the RANKL/OPG ratio was investigated, resulting in a good positive correlation, as shown in Figure 4d.

Monitoring SuPAR and sCD14ST levels may offer valuable insights into immune system dysregulation and bone-related complications in conditions characterized by inflammation. These soluble receptors represent important links between immune activation and bone metabolism, especially in the context of diseases like COVID-19, where the inflammatory response may impact bone fragility.

### Limitations

This study is an observational one and has some limitations, such as the number of patients and the time points evaluated. The low numbers of COVID-19 patients results from the challenge of recruiting COVID-19-positive patients undergoing orthopedic surgery. The kind of surgery considered in this study was not emergency surgery, so it could be delayed for a long time in the case of infection. In this study, patient enrollment was performed in the late phase of COVID-19 infection, when the recommendation of the Ministry of Health was to avoid undertaking any non-emergency surgical intervention on COVID-19-positive patients in order to prevent the spread of the infection in hospitals. Therefore, the enrollment of COVID-19-positive patients was very challenging and could be based only on those patients undergoing programmed surgery who were incidentally determined to be positive at the pre-preoperatory routine test and who could not delay the orthopedical surgery.

Another limitation is the longitudinal extension of the study. Since this is an observational study, the time points considered were evaluated according to the orthopedic surgery clinical protocol, in agreement with the Ethical Committee. An extended longitudinal evaluation may potentially increase the impact of the longitudinal value of these biomarkers, which will be considered in the future development of this study.

## 5. Conclusions

The intersection of COVID-19 and osteoimmunology highlights the complex relationship between the immune system and bone health. As research into osteoimmunology improves, a better understanding of these mechanisms may provide opportunities for improving bone health during and after COVID-19, as well as offering therapeutic strategies to mitigate bone-related complications in these patients. In this context, the study of sCD14ST and its connection to osteoimmunology highlights the critical role of the immune system in regulating bone health. Understanding how sCD14ST influences immune activation, osteoclastogenesis, and bone remodeling opens up potential therapeutic avenues for treating inflammatory bone diseases. By targeting immune pathways involving sCD14ST, we may be able to mitigate the effects of chronic inflammation on bone health.

## Data Availability

Data are unavailable due to privacy restrictions.
